# Psychological Resilience and Associated Factors in Cancer Patients: A Cross‐Sectional Analysis

**DOI:** 10.1002/cnr2.70415

**Published:** 2026-01-02

**Authors:** Marta Esteban Blanco, Inmaculada Martinez de la Viuda, Maria Gemma Rodriguez Chico, Maria del Mar Félix Montalvo, Aurea Garcia Salas, Andrés Garcia Palomo, Maria Teresa Quintana Asenjo

**Affiliations:** ^1^ Instituto de Investigación Biosanitaria de León (IBIOLEÓN) León Spain; ^2^ Department of Nurse Oncology Hospital of León León Spain; ^3^ Department of Oncology Hospital of León León Spain

**Keywords:** anxiety, Apgar, depression, illness, mental health, oncology, psychological, quality of life, resilience, social support

## Abstract

**Background:**

Cancer remains one of the leading global health problems, with treatments that can compromise patients' quality of life.

**Aims:**

This study aimed to determine the prevalence and characteristics of resilience in cancer patients and to analyze the influence of psychological and social factors on disease perception.

**Methods and Results:**

An analytical cross‐sectional study was conducted between April and August 2023 including 61 cancer patients under treatment. Resilience was assessed with the RS‐14, anxiety and depression with HADS (Hospital Anxiety and Depression Scale), and family functioning with the Family Apgar (Adaptation, Partnership, Growth, Affection, Resolve questionnaire). Sociodemographic and clinical data were obtained through structured questionnaires. Continuous variables were tested with Shapiro–Wilk and Levene's tests; descriptive statistics, t‐tests, Mann–Whitney U, Chi‐square were applied. Binary logistic regression examined resilience predictors, adjusting for confounders (BMI, employment status, surgery). Statistical significance was defined as *p* ⟨ 0.05. Participants were 50.8% women, aged 35–82 years. Cancer types included breast (18%), lung (29.5%), colon (9.8%), pancreas (11.5%), renal (1.6%), and others (29.5%). 11.5% had not received oncological treatment, while 93.4% underwent surgery. Most were non‐smokers (82%) and retired (57.4%). Main comorbidities were respiratory (24.6%) and cardiovascular (23%). In surveys, 54.1% reported family members with cancer and 36.1% noted a lack of free time affected quality of life. Mean scores: resilience 69.3 (SD = 22.1), anxiety 10.3 (SD = 3.2), depression 11.6 (SD = 2.2), Apgar 17.1 (SD = 3.7). Logistic regression identified Apgar as the only significant predictor of resilience (OR = 0.294, 95% CI 0.113–0.761, *p* = 0.012), with higher family functioning linked to lower resilience. Model accuracy was 81.1% overall, 90.9% for resilient, and 65.0% for non‐resilient patients.

**Conclusions:**

Social, clinical, and family situations all have an impact on cancer patients' resilience. In order to maximize resilience and quality of life, family functioning appears as a contradictory component, indicating the necessity of psychological, family‐centered, and interdisciplinary interventions.

**Abbreviations**
APGAR: Adaptation, Partnership, Growth, Affection, Resolve questionnaire
BMI: body mass index
CEIm: Committee on Ethics for Drug Research
CI: confidence interval
HADS: Hospital Anxiety and Depression Scale
N/A: not available
OR: odds ratio
RS‐14: Resilience Scale—14 items
SD: standard deviation
WHO: World Health Organization

## 1 Introduction

Cancer is one of the main public health problems worldwide [1]. Nowadays, the word “cancer” is associated with the threat of life, death and even evasion of the name of this disease. In addition, patients should consider different treatment methods, such as surgery, radiation, and/or chemotherapy [2] and could experience long‐term physical symptoms due to complications from surgery and side effects from medications [3]. Even when treatment is effective, patients continue to experience anxiety about relapse and potential complications. The different methods of treatment and care for cancer patients, including the amount of medication, can disrupt daily life and affect their quality of life [2, 4, 5].

Previous studies inform us that the incidence of anxiety and depression among cancer patients is higher than in the general population [6, 7]. A descriptive study in women undergoing breast cancer surgery reported an anxiety prevalence of 17.9% [8]. A systematic review in multiple geographical regions and various cancer types, including breast, digestive tract, lung and other respiratory, hematological, head and neck, brain, skin, genitourinary, endocrine, bone and soft tissue malignancies, as well as mixed groups, showed that depression prevalence ranged from 7% in patients with skin cancer to 31% in those with digestive tract cancer when assessed with self‐report tools, and from 3% in lung cancer to 28% in brain cancer when measured with diagnostic interviews [9]. Moreover, depression prevalence was highest during the acute treatment phase (14%–27%), compared to lower rates during the first year post‐treatment (9%–21%) and after 1 year (8%–15%) [9]. Anxiety and depression cause failures in medical consultations, adherence to treatment and also in quality of life [10].

The World Health Organization (WHO) defines quality of life as the subjective perception that an individual has of their position in life, in a cultural framework and in a set of values in which they live, in relation to their objectives, expectations and concerns [4]. Moreover, with the rising costs of health care and growing concerns about the effectiveness of treatment interventions, quality of life has recently emerged as a key objective in the management of chronic diseases [11].

Also, according to positive psychology, certain elements increase compatibility with life's demands and risks [12]. For example, cancer patients need to learn many skills to self‐manage their symptoms (such as nausea, pain, fatigue) and deal with ongoing intrusive thoughts about possible death during treatment [13].

Psychological resilience has been broadly defined as the ability to adapt to stressors, recover from adversities and life crises, and resume normal psychosocial functioning soon after adverse events [14], it may depend on several factors, including positive emotions, cognitive flexibility (such as acceptance), active coping style and spirituality, and so forth. Resilience has been shown to play a protective role against distress in cancer patients [15, 16, 17, 18].

Studies in cancer patients show high levels of resilience that help people use positive emotions to overcome unpleasant experiences and improve their quality of life. For example, a study by Gotay et al. in patients with breast, stomach and lung cancer showed that resilience is related to a better quality of life and low levels of depression [19]. Another study examined pain in cancer patients receiving radiation therapy and the results showed that resilience is a strong predictor of quality of life and adaptability in patients [20].

If the improvement of psychological well‐being is considered an end point of medical care along with survival, mortality and morbidity, then resilience could be considered a support between cancer symptoms and patient distress [21].

The hypothesis we propose is that higher resilience levels in cancer patients are positively associated with better family functioning (measured by the Apgar score: Adaptation, Partnership, Growth, Affection, Resolve questionnaire) and lower levels of anxiety and depression.

Therefore, the main objective of our study is to know the resilience capacity of the cancer patient hospitalized in our center. Also, the specific objectives are to study the prevalence and characteristics of resilience in cancer patients and the psychological and social factors that could influence the perception of the disease.

## 2 Materials and Methods

### 2.1 Ethics

The study was conducted at the Hospital Princesa Sofía de León (Spain) and was approved by the Ethics Committee for Research with Medicinal Products of the Health Areas of León and El Bierzo (CEIm code: 22139), which oversees the ethical review of research conducted within the healthcare area that includes Hospital Princesa Sofía de León. All participants provided written informed consent prior to inclusion, in accordance with the principles of the Declaration of Helsinki.

### 2.2 Study Design and Participants

This is an analytical cross‐sectional study conducted between April and August 2023 at the Princess Sofía Hospital of León, Spain. Each patient must meet all the inclusion criteria and none of the exclusion criteria to be able to take part in the study. Inclusion criteria were: to be over 18 years old, to suffer or have suffered from a cancer diagnosis, and to be admitted to the León hospital and patients who provide their written informed consent. On the contrary, exclusion criteria were to be under 18 years old and to have never been diagnosed with cancer or patients who, due to their level of consciousness at the time of the study, are not available and thus make it difficult for them to complete the questionnaires.

**FIGURE 1:**
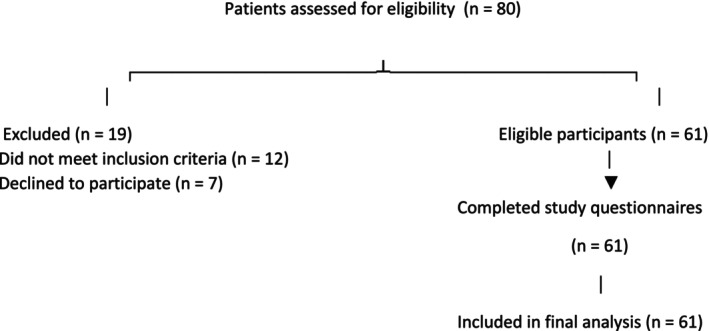
Flow diagram of patient inclusion and exclusion in the study.

Participants being diagnosed for different types of cancer (*n* = 80) and all of them undergoing oncological treatment (chemotherapy, radiotherapy…) were recruited (Figure 1).

### 2.3 Sample Size and Sampling Strategy

Participants were chosen using a non‐probability purposive sampling technique. A cross‐sectional study's sample size was determined with a 95% confidence level and 5% precision. Since no previous local data were available, a cautious estimate of 50% for the estimated prevalence of moderate‐to‐high resilience was employed to ensure a sufficiently powered sample. Based on this calculation, a minimum of 60 participants was required. During the study period (April–August 2023), 80 patients were screened for eligibility, of whom 61 met the inclusion criteria, provided written informed consent, and completed the study questionnaires administered by research nurse assistants. With this strategy, the recruited sample was guaranteed to satisfy the minimal calculated need and accurately reflect the study population.

### 2.4 Outcome Variable and Instruments

A specifically designed socio‐demographic questionnaire was used to collect information on: Age (continuous numerical variable, measured in years); Gender (nominal categorical variable: male, female,); Employment status (nominal categorical variable: study, paid work, retired, inability, others, retired, etc.); Body Mass Index (BMI) (continuous numerical variable, measured in kg/m^2^); Allergies (nominal categorical variable: yes/no); Smoking habits (nominal categorical variable: yes/no quantified by cigarettes/day); Comorbidities (nominal categorical variable, list of present conditions); Type of treatment (nominal categorical variable: chemotherapy, radiotherapy, immunotherapy, combination, or no treatment: yes/no); and Cancer diagnosis (nominal categorical variable: type of cancer: breast, lung, colon, pancreas, renal, other).

The primary outcome was resilience, assessed using the Resilience Scale RS‐14 (Resilience Scale–14 items) developed by Wagnild and Young [22]. The Spanish version consists of 14 items scored on a 7‐point Likert scale (1 = “strongly disagree” to 7 = “strongly agree”), yielding a total score between 14 and 98 points, with scores ≥ 70 indicating high resilience. The scale has demonstrated good validity and reliability across various clinical contexts [23].

Secondary variables included anxiety and depression, measured by the Hospital Anxiety and Depression Scale (HADS), developed by Zigmond and Snaith in 1983 [24], is a self‐assessment questionnaire designed to detect and quantify levels of anxiety and depression in hospital and outpatient settings. Its primary benefit is its emphasis on psychological expressions of anxiety and sadness, hence reducing the impact of physical symptoms brought on by medical sickness. The scale consists of 14 items, divided into two subdomains: anxiety (HADS‐A, 7 items) and depression (HADS‐D, 7 items). Each item evaluates the severity or frequency of symptoms experienced over the past week. Items are scored on a 4‐point scale from 0 to 3, with higher scores indicating greater symptom severity. Scores for each subdomain range from 0 to 21, and are interpreted as follows: 0–7 = normal, 8–10 = borderline or possible case, and 11–21 = probable case of anxiety or depression. While the total HADS score ranges from 0 to 42, it is common practice to interpret the anxiety and depression subdomains independently. The questionnaire is brief, easy to administer, and does not require specialized training.

Apgar questionnaire (Adaptation, Partnership, Growth, Affection, Resolve) developed by Smilkstein et al. [25], analyzes the family support perceived by participants. This family questionnaire can be used to show how an individual views the way their family is operating at a particular moment. In contexts like outpatient settings, where it is uncommon for patients or users to openly discuss family issues, documenting this impression is very crucial. The scale consists of five items. In each item, there are three options to measure the feeling of satisfaction Each response has a score ranging from 0 to 4 points, according to the following rating: 0 (never), 1 (almost never), 2 (sometimes), 3 (almost always), and 4 (always). Score interpretation: Normal: 17–20 points, Mild dysfunction: 16–13 points, Moderate dysfunction: 12–10 points, Severe dysfunction: less than or equal to 9.

Additionally, four general questions were administered to explore patients' perceived health status and quality of life.

### 2.5 Potential Sources of Bias

To minimize selection bias, all eligible patients during the study period were approached consecutively, ensuring that no subgroup of patients was systematically excluded. Information bias was reduced by using validated and standardized instruments for all primary and secondary outcomes, such as the HADS for anxiety and depression, the RS‐14 for resilience, and the Apgar questionnaire for family functioning. Study nurses only assisted when necessary to answer questions without influencing responses, and patients received consistent instructions on how to complete the questionnaire. Confounding bias was addressed in the statistical analysis by adjusting logistic regression models for potential confounders such as age, sex, and BMI. In order to prevent bias from incomplete responses, missing data were also addressed using multiple imputation for regression models and pairwise deletion for descriptive and correlation analyses.

### 2.6 Statistical Analysis

All statistical analyses were conducted using SPSS version 22.0. Continuous variables were assessed for normality using the Shapiro–Wilk test and for homogeneity of variances using Levene's test. Descriptive statistics are presented as means and standard deviations for normally distributed variables, and as medians and interquartile ranges for non‐normally distributed variables. Categorical variables are reported as frequencies and percentages. Inferential analyses were conducted using Student's *t*‐test for normally distributed continuous variables, Mann–Whitney *U* test for non‐normally distributed continuous variables, and Chi‐square test for categorical variables. Adjusted logistic regression models were performed to examine the relationship between resilience (outcome) and predictor variables. The models included relevant confounders (BMI, Surgery, and employment) to control for potential bias. Odds ratios (ORs) and 95% confidence intervals (CIs) were reported. Missing data were handled by pairwise deletion for descriptive and correlation analyses and by multiple imputation for regression models. Differences were regarded as statistically significant when the *p* value was below 0.05.

## 3 Results

### 3.1 Sociodemographic Data and Descriptive Statistics

Participants that finally complete the questionnaires (*n* = 61) were adults with a range age between 35 and 82 years old and 50.8% of them were women (Table 1). The majority were non‐smokers (82%). All of them had been diagnosed with different types of cancer (breast cancer [18.0%], lung [29.5%], colon [9.8%], pancreas [11.5%], renal [1.6%], and others [29.5%]). 11.5% had not received adjuvant treatment; in addition, surgery was present in 93.4% of patients. Most of the participants were not smoking (82.0%). In the professional area, 57.4% of them were retired, 19.6% were working, and 18% were in a situation of inability.

**TABLE 1:** Socio‐demographic and clinical variables for the oncological sample.

Socio‐demographic variables		Total (*n* = 61)		Clinical variables		Total (*n* = 61)	
		*n*	%			*n*	%
Age (years)	35–82			Smoke	Yes	11	18
					No	50	82
Gender (%)	Woman	31	50.8	Stages	In progress	57	93.4
	Man	30	49.2		Complete	4	6.6
Allergic	Yes	9	13.1	Surgery	Not surgery	17	27.87
	No	52	83.6		Biopsy only	21	34.43
Employment (%)	Studying	1	1.6		Total resection only	8	13.11
					Multiple surgeries	11	18.03
	Paid work	11	18		Others	4	6.56
	Retired	35	57.4				
	Inability	11	18				
	Others	3	4.9				

*Note:* *n* = simple size.

The frequency of the different comorbidities suffered by the patients in the study was 24.6% respiratory, 23% cardiovascular, 9.8% hepatic, 14.8% digestive, 13.1% locomotive, 1.6% renal, 18% psychiatric/psychological and 3.3% hematological comorbidity.

We also conducted a survey with 4 questions that we found interesting in order to analyze the patient's life more thoroughly, 54.1% of patients answered affirmatively to the first question “Is there anyone in your close family with cancer problems?”, 21.31% answered affirmatively to the second question “Is there someone in your close family with a serious illness?”, to the question “During the last 4 weeks, how many days did you have to miss work or school because of illness?” The patients responded: 32.8% negatively, 3.3% between 1 and 7 days, 3.3% between 15 and 21 days, and 19.7% more than 21 days. The last question “Do you consider that any of these issues could be affecting your quality of life?” is divided into several questions: My relationship with my teachers or bosses 1.6% answered affirmatively, my academic results 11.5% answered affirmatively/my work achievements 6.7% answered affirmatively, the lack of free time 36.1% answered affirmatively, my relationship with my family 37.7% answered affirmatively (Table 2).

**TABLE 2:** General questions about the health status and quality of life for the patients on this study.

Questions		Total (*n* = 61)	
		*n*	%
Is there anyone in your close family with cancer problems?	No	28	45.9
	Yes	33	54.1
Is there someone in your close family with a serious illness?	No	43	70.49
	Yes	13	21.31
	N/A	5	8.20
During the last 4 weeks, how many days did you have to miss work or school because of illness?	No	20	32.79
	1–7 days	2	3.28
	15–21 days	2	3.28
	More 21 days	12	19.67
	N/A	25	40.98
Do you consider that any of these issues could be affecting your quality of life?			
My relationship with my teachers or bosses	No	28	45.90
	Yes	1	1.64
	N/A	32	52.46
My academic results	No	21	34.43
	Yes	7	11.48
	N/A	33	54.10
My work achievements	No	25	40.98
	Yes	4	6.56
	N/A	32	52.46
The lack of free time	No	19	31.15
	Yes	22	36.07
	N/A	20	32.79
My relationship with my family	No	27	44.26
	Yes	23	37.70
	N/A	11	18.03

*Note:* N/A: Not available; *n* = sample size.

Descriptive statistics were calculated for the main variables. Average scores were 69.31 (SD = 22.14) for resilience, 10.25 (SD = 3.19) for anxiety, 11.57 (SD = 2.24) for depression, and 17.14 (SD = 3.68) for Apgar score.

### 3.2 Association Analysis

An analysis of potential confounding variables was initially conducted to identify those that might influence the relationship between the main predictors (anxiety, depression, and Apgar) and resilience. The results are presented in Table 3, which shows the simple coefficients, the adjusted coefficients after including each variable, the percentage change in the coefficients, and the determination of whether the variable acts as a confounder. The variables identified as confounders were BMI, employment status, and surgery, as their inclusion modified the coefficients of the main predictors by more than 10%, indicating that they could bias the estimate of the effect of anxiety, depression, or Apgar. Other variables such as sex, smoking, tumor type, and stage were not classified as confounders.

**TABLE 3:** Analysis of the confounding variables of the study on Resilience.

	Independent variable	Anxiety	Depression	Apgar
Sex	OR simple	−0.274	0.369	−0.24
	OR adjusted	−0.293	0.371	−0.241
	% change	6.9	0.5	0.4
	Confusor?	No	No	No
BMI	OR adjusted	−0.309	0.402	−0.265
	% change	12.8	8.9	10.4
	Confusor?	Yes	No	Yes
Smoke	OR adjusted	−0.289	0.358	−0.246
	% change	5.5	−3.0	2.5
	Confusor?	No	No	No
Type of tumor	OR adjusted	−0.281	0.37	−0.242
	% change	2.6	0.3	0.8
	Confusor?	No	No	No
Stage	OR adjusted	−0.271	0.368	−0.255
	% change	−1.1	−0.3	6.3
	Confusor?	No	No	No
Employment	OR adjusted	−0.322	0.321	−0.212
	% change	17.5	−13.0	−11.7
	Confusor?	Yes	Yes	Yes
Surgery	OR adjusted	−0.303	0.314	−0.224
	% change	10.6	−14.9	−6.7
	Confusor?	Yes	Yes	No

Abbreviation: OR: odds ratio.

Based on this analysis, an adjusted logistic regression model was constructed, including Anxiety, depression, and Apgar as primary predictors, and BMI, surgery and employment status as confounders. The results of the adjusted model are shown in Table 4.

**TABLE 4:** Logistic regression: OR, 95% CI and significance of the variables.

Variable	OR	Sig.	95% CI	
			Lower	Upper
Anxiety	0.243	0.067	0.053	1.107
Depression	3.383	0.145	0.658	17.398
Apgar	0.294	0.012	0.113	0.761
BMI	1.204	0.146	0.937	1.547
Employment	0.700	0.330	0.341	1.436
Surgery	1.287	0.467	0.652	2.540

Abbreviations: CI: confidence interval; OR: odds ratio; Sig: level of significance.

A binary logistic regression model was constructed to predict resilience based on six independent variables: anxiety, depression, Apgar, BMI, employment status, and Surgery. In the analysis, Apgar was the only variable significantly associated with resilience (OR = −1.225; Exp(OR) = 0.294; 95% CI 0.113–0.761; *p* = 0.012), indicating that higher Apgar scores are associated with a lower likelihood of resilience.

The BMI, employment and surgery variables were included as confounders, as their inclusion allows for controlling for potential bias and ensures that the Apgar effect estimate accurately reflects its true effect. In the model, BMI showed a slight positive trend on resilience (OR = 0.186; Exp(OR) = 1.204; *p* = 0.146), indicating that for each additional unit of BMI, the probability of resilience tends to increase by 20.4%. Employment status showed a negative trend (OR = −0.357; Exp(OR) = 0.700; *p* = 0.330), suggesting that certain changes in employment status are associated with an approximate 30% reduction in the probability of resilience. Finally, surgery showed a slight positive trend (OR = 0.252; Exp(B) = 1.287; *p* = 0.467), indicating that for each additional unit of surgery, the probability of resilience tends to increase by 28.7%.

The model's accuracy was assessed using the classification table, which indicated that the model correctly predicted 81.1% of cases overall, with an accuracy of 90.9% for resilient subjects and 65.0% for non‐resilient subjects, thus demonstrating adequate predictive capacity, especially for identifying resilient individuals. In this study, the prevalence of resilience among hospitalized cancer patients was relatively high, with a majority of participants classified as resilient according to the RS‐14 scale.

## 4 Discussion

The outcomes of the study provide relevant insights into the psychosocial dynamics of resilience in cancer patients. Unlike previous expectations, our logistic regression analysis showed that, among the variables examined, the only factor significantly associated with resilience was family functioning as measured by the Family Apgar questionnaire. Specifically, higher Apgar scores were linked to a lower probability of resilience, a result that requires careful interpretation given the established role of family support in oncological adjustment. In addition, BMI, employment status, and surgery acted as confounders, influencing the associations between psychological variables and resilience.

These findings contrast with a large body of literature that has consistently emphasized resilience as a protective psychological factor, closely linked to reduced levels of anxiety and depression in cancer patients. In our descriptive analysis, average levels of anxiety and depression were moderate, and simple correlations initially suggested associations between these variables and resilience. However, once potential confounders were controlled for, the significance of anxiety and depression diminished. This suggests that the psychological adjustment of oncology patients cannot be explained solely by intrapsychic factors, but is instead shaped by a complex interplay of clinical and sociodemographic conditions [19, 20, 21].

One of the most notable aspects of this study is the role of the Apgar score. Traditionally, family support has been described as a central resource in improving psychological well‐being and resilience in patients facing cancer [25, 26]. Supportive family environments provide emotional security, enhance adherence to treatments, and improve quality of life [27, 28]. For this reason, our finding that higher Apgar scores predicted lower resilience may appear counterintuitive. A possible explanation is that patients with strong family functioning may rely more heavily on their family as a coping mechanism, which could reduce the necessity for developing high levels of individual resilience. In contrast, patients with less supportive family environments may be forced to cultivate greater personal resilience as a compensatory mechanism. This interpretation aligns with theoretical models suggesting that resilience is a dynamic adaptation process, influenced both by internal capacities and external resources [28, 29, 30].

The inclusion of BMI, employment status, and surgery as confounders highlights the relevance of contextual and physical health variables in resilience research. In our study, higher BMI showed a positive tendency toward resilience, although it did not reach statistical significance. This result could be understood in the context of prior findings linking physical condition and activity levels with psychological well‐being in cancer patients [26]. Employment status, on the other hand, showed a negative trend, suggesting that occupational instability or inability to work may undermine patients' psychological adjustment. Employment has often been described as a source of social identity and daily structure; thus, its loss may create additional stressors that interact with resilience [21, 27]. Finally, surgery appeared to have a slight positive association with resilience, which could reflect the perception of surgery as a decisive treatment measure, potentially offering patients a sense of progress or control over their illness.

It is important to note that the predictive model used in this study demonstrated good accuracy, correctly classifying over 80% of participants, with particularly strong predictive power for resilient individuals. This reinforces the validity of our findings and underscores the utility of logistic regression for disentangling complex psychosocial interactions in oncology research.

The outcomes of the study provide significant information about the psychosocial dynamics of cancer patients' resistance. Contrary to expectations, our logistic regression analysis revealed that family functioning as assessed by the Family Apgar questionnaire was the only variable among those analyzed that was substantially linked to resilience. Specifically, higher Apgar scores were linked to a lower probability of resilience, a result that requires careful interpretation given the established role of family support in oncological adjustment. In addition, confounders such as BMI, work status, and surgery also affected the relationships between resilience and psychological factors.

These findings contrast with a large body of literature that has consistently emphasized resilience as a protective psychological factor, closely linked to reduced levels of anxiety and depression in cancer patients. Simple correlations first indicated links between anxiety and depression and resilience, and average levels of both were moderate in our descriptive study. However, the importance of anxiety and sadness decreased after possible confounders were taken into account. This suggests that the psychological adjustment of oncology patients cannot be explained solely by intrapsychic factors, but is instead shaped by a complex interplay of clinical and sociodemographic conditions [19, 20, 21].

One of the most notable aspects of this study is the role of the Apgar score. Traditionally, family support has been described as a central resource in improving psychological well‐being and resilience in patients facing cancer [25, 26]. Supportive family environments provide emotional security, enhance adherence to treatments, and improve quality of life [27, 28]. For this reason, our finding that lesser resilience was predicted by higher Apgar scores may seem contradictory because of this. One possibility is that patients who have great family functioning might use their family more frequently as a coping strategy, which might lessen the need for them to build strong personal resilience. As a compensating approach, patients with less supportive home settings might be compelled to develop higher personal resilience. This interpretation aligns with theoretical models suggesting that resilience is a dynamic adaptation process, influenced both by internal capacities and external resources [28, 29, 30].

The fact that BMI, work status, and surgery are included as confounders emphasizes how important physical and contextual health factors are in resilience studies. In our study, higher BMI showed a positive tendency toward resilience, although it did not reach statistical significance. This result could be understood in the context of prior findings linking physical condition and activity levels with psychological well‐being in cancer patients [26]. Employment status, on the other hand, showed a negative trend, suggesting that occupational instability or inability to work may undermine patients' psychological adjustment. Employment has often been described as a source of social identity and daily structure; thus, its loss may create additional stressors that interact with resilience [21, 27]. Finally, surgery appeared to have a slight positive association with resilience, which could reflect the perception of surgery as a decisive treatment measure, potentially offering patients a sense of progress or control over their illness.

It is important to note that the predictive model used in this study demonstrated good accuracy, correctly classifying over 80% of participants, with particularly strong predictive power for resilient individuals. This reinforces the validity of our findings and underscores the utility of logistic regression for disentangling complex psychosocial interactions in oncology research.

Recent studies highlight the potential role of non‐clinical digital health (eHealth) solutions in promoting cancer patients' mental health in addition to psychological and family‐focused therapy. According to a recent systematic review, a variety of digital interventions, such as web‐based platforms, mobile apps, and remote support tools, have demonstrated encouraging outcomes in reducing anxiety, improving emotional coping, and improving the perceived quality of life for patients and caregivers. The observed relationships between resilience, anxiety, and family functioning suggest that incorporating eHealth strategies could supplement traditional psychological care by offering easily accessible resources for self‐management and emotional support, even though the current study did not specifically look at these interventions [31]. In order to maximize resilience and mental health outcomes in oncology settings, future research should examine how combining these digital tools with conventional psychosocial therapy could be implemented.

In summary, this study demonstrates that resilience in cancer patients is not determined only by psychological variables such as anxiety or depression but is also influenced by social and clinical contexts. The important but complicated role of family functioning is highlighted by the strong correlation with Apgar. These results provide credence to the development of integrated psychological interventions that enhance relational and personal resources while also investigating cutting‐edge instruments like digital health solutions to supplement established support networks. Future research should continue to investigate how these factors interact dynamically, with the goal of optimizing resilience and, consequently, the quality of life of oncology patients.

## 5 Limitations

In our study, we detected a selection bias because some patients were suffering from severe cases of cancer, and their overall condition was significantly deteriorating. As a result, they declined to participate in the study.

A temporal association between the exposure and the result could not be established because of the cross‐sectional design of this study. To investigate the predictive effects of the primary variables on health outcomes, a longitudinal analysis might be relevant.

The participants had different types of cancer, which could lead to the results not being generalizable and could affect the resilience of the patients. For future studies, it would be recommendable to try to increase sample homogeneity with respect to clinical variables such as diagnosis, subsequent survival, and so forth.

Furthermore, the sample size was somewhat small, which might have limited the findings' robustness and statistical power. To improve the reliability and generalizability of the findings, future studies should try to enlist a bigger and more representative patient group, maybe through multi‐center collaborations.

## 6 Clinical Implications

The findings of this study have several relevant implications for clinical practice. First, they highlight the need to adopt a multifactorial perspective when addressing resilience in oncology patients. While psychological variables such as anxiety and depression are important and should be properly assessed and treated, they do not appear to fully explain resilience levels. A thorough assessment should take into account social, familial, and clinical elements that affect resilience, such as family functioning, work status, or surgical procedures.

Second, our results emphasize the complex role of family support. Although the literature has consistently pointed out that support networks are fundamental for psychological well‐being, in this study higher family functioning was associated with a lower need for individual resilience. This does not imply that family support is negative, but rather that it may modify the way resilience manifests, shifting the balance between personal and collective coping resources. Therefore, clinical interventions should support individual coping mechanisms that allow patients to acquire emotional autonomy and personal adaptation methods, in addition to encouraging family support and support group participation.

Third, integrating particular psychological interventions, like cognitive‐behavioral therapy, can assist in managing depression, lessen anxiety symptoms, and ultimately promote resilience. These interventions should be complemented by strategies that promote self‐reflection and emotional self‐management, enhancing patients' ability to recognize their emotions, ask for help, and actively cope with the difficulties associated with cancer.

Finally, the study reinforces the need for a multidisciplinary approach in oncology, where physicians, psychologists, social workers, and other professionals collaborate in a coordinated way. In addition to making it possible to identify patients who are more emotionally vulnerable, this kind of collaboration facilitates the development of specialized support plans that incorporate clinical resources and advancements in digital health. In this way, it is possible to strengthen patients' resilience and significantly improve their quality of life during the oncological process.

## 7 Conclusions

Resilience in cancer patients is shaped by psychological, family, social, and clinical factors. Family functioning plays a complex role, influencing how individual resilience develops. BMI, employment status, and surgery also impact resilience and should be considered in assessments. Clinically, interventions should combine psychological support, family‐centered approaches, and digital health tools. A multidisciplinary approach is essential to identify vulnerable patients and promote adaptive coping. Future research should explore these interactions longitudinally to enhance personalized resilience interventions and improve quality of life.

## Author Contributions

**Marta Esteban Blanco:** data curation (lead), formal analysis (lead), methodology (lead), project administration (lead), writing – original draft (lead), writing – review and editing (lead). **Inmaculada Martinez de la Viuda:** conceptualization (equal), investigation (equal), resources (equal), validation (equal). **Maria Gemma Rodriguez Chico:** conceptualization (equal), investigation (equal), resources (equal), validation (equal). **Maria del Mar Félix Montalvo:** conceptualization (equal), investigation (equal), resources (equal), validation (equal). **Aurea Garcia Salas:** conceptualization (equal), investigation (equal), resources (equal), validation (equal). **Andrés Garcia Palomo:** conceptualization (equal), funding acquisition (lead), investigation (equal), resources (equal), validation (equal). **Maria Teresa Quintana Asenjo:** conceptualization (equal), investigation (equal), resources (equal), supervision (lead), validation (equal).

## Conflicts of Interest

The authors declare no conflicts of interest.

## Data Availability

The data that support the findings of this study are available on request from the corresponding author. The data are not publicly available due to privacy or ethical restrictions.
